# Nitrogen form regulates ginsenoside biosynthesis in *Panax ginseng* adventitious roots: comparative transcriptomics of ammonium- and nitrate-mediated pathways

**DOI:** 10.3389/fpls.2026.1948127

**Published:** 2026-09-28

**Authors:** Siyu Zhang, Qi Yuan, Ruyue Qiu, Ri Gao, Songquan Wu, Xiangguo Li

**Affiliations:** Yanbian University, Yanji, China

**Keywords:** adventitious roots, ammonium nitrogen, nitrate nitrogen, *Panax ginseng*, transcriptome

## Abstract

*Panax ginseng* C. A. Mey. is a valuable Chinese herbal medicine. Saponins are one of its main active ingredients, possessing various important pharmacological activities and serving as key indicators for evaluating ginseng quality. Due to the depletion of wild ginseng resources, adventitious root culture has emerged as an effective approach for ginsenoside production; however, the ginsenoside content in cultured adventitious roots remains relatively low. Nitrogen, as an essential macronutrient for plant growth and development, plays a vital role in both biomass accumulation and secondary metabolite biosynthesis. Different nitrogen forms exert distinct regulatory effects on the synthesis of triterpenes, but the molecular mechanism of ammonium nitrogen and nitrate nitrogen regulating the accumulation of ginsenosides in ginseng adventitious roots is not clear. In this study, we combined physiological measurements with comparative transcriptomic analysis to investigate the regulatory pathways underlying adventitious root growth and differential ginsenoside accumulation under nitrate-only, ammonium-only, and nitrogen-free conditions at the optimal nitrogen concentration. Physiological results showed that nitrate nitrogen supply significantly increased adventitious root biomass and total ginsenoside content compared with ammonium nitrogen. Functional enrichment analysis of transcriptomic data revealed significant transcriptional remodeling in terpenoid biosynthesis, plant hormone signal transduction, and redox homeostasis pathways. Further analysis showed that the core genes of ginsenoside synthesis pathway were upregulated under nitrate nitrogen conditions. Nitrate treatment also resulted in higher levels of endogenous IAA and CTK and activation of most hormone signaling pathways, whereas ammonium nitrogen induced a significant increase in CAT and POD antioxidant enzyme activities. Nitrate nitrogen can promote endogenous nitric oxide (NO), and further pharmacological experiments suggest that NO likely acts as an important intermediate signal contributing to nitrate-driven promotion of adventitious root growth and ginsenoside synthesis. The results clarify the molecular mechanism of nitrogen form regulating ginseng secondary metabolism and provide a theoretical basis for optimizing the nitrogen strategy of ginseng adventitious root culture and improving the yield of ginsenosides.

## 1 Introduction

*Panax ginseng* C. A. Mey. is a perennial herb belonging to the family Araliaceae. Its medicinal parts include dry roots and rhizomes. Ginseng is a well-known traditional Chinese medicinal resource with medicinal, edible, and health-care properties and is considered to have nutritional and pharmacological potential (Zhan et al., 2014; Tao et al., 2023; Song et al., 2026). Ginseng has been reported to possess various pharmacological effects, including anticancer, antiaging, and immunity-enhancing activities (Ru et al., 2015; Kim, 2018; Zhang et al., 2026). Ginseng contains saponins, polysaccharides, peptides, fatty acids, sterols, alkaloids, lignins, flavonoids and other ingredients (Huang et al., 2023). Of these, ginsenosides are regarded as an important index to evaluate ginseng quality. Ginsenoside, a triterpenoid, is the main secondary metabolite of ginseng and widely recognized as an important bioactive compound (Shin et al., 2015; Chen et al., 2025). Since the 1960s, researchers have isolated and identified more than 100 ginsenosides from various ginseng tissues (Kim et al., 2014; Ru et al., 2015). Ginsenosides are mainly found in ginseng. Based on their different glycosidic bonds, they are divided into protopanaxadiol type ginsenosides (PPD type: Rb1, Rb2, Rc, and Rd), protopanaxatriol type ginsenosides (PPT type: Re, Rf, and Rg1) (Christensen, 2008), and oleanolic acid type ginsenosides (Ro). Of these, PPD and PPT-type ginsenosides are dammarane-type tetracyclic triterpenoids composed of isoprene units, accounting for more than 80% of the total ginsenosides; oleanolic acid-type ginsenosides are pentacyclic triterpenoids, and only ginsenoside Ro has been identified (Shin et al., 2015). The biosynthesis of ginsenosides involves a multi-step enzymatic cascade, in which key enzymes such as HMGR, FPS, SQS, SQE, DDS, β-AS, CYP450, and GT collaboratively regulate the efficiency, skeleton formation, and final structural diversity of ginsenosides (Wu et al., 2010; Kim et al., 2014).

Compared to field soil cultivation, adventitious root culture offers advantages, such as easy environmental control, a short growth period, and strong genetic stability, making it a promising technological approach for the production of high-value secondary metabolites (Murthy et al., 2014; Zhang et al., 2022). This technology has been successfully applied on an industrial scale for biomass and bioactive compound production from various medicinal plants, including *P. ginseng* (Devi et al., 2021) and *Polygonatum multiflorum* (Ho et al., 2021). For instance, Jiang et al. (2015) cultured the adventitious roots of *Oplopanax elatus* in a balloon-type bioreactor and obtained polysaccharide content comparable to that of three-year-old field-grown plants. Cui et al. (2011) used a balloon-type bioreactor to cultivate adventitious roots of *Forsythia suspensa* for the production of phenolic and flavonoid compounds. Zhao et al. (2020) established a stable and efficient adventitious root proliferation system for Panax notoginseng, and found that its ginsenoside Re content was 1.8 times that of three-year-old, field-grown plants. Yin et al. (2014) investigated the effects of the ammonium/nitrate ratio and phosphate concentration on biomass and metabolite accumulation in adventitious roots of *Glycyrrhiza uralensis* and determined that the optimal ammonium/nitrate ratio for glycyrrhetinic acid production was 10:20.

Nitrogen (N) is an essential macronutrient that profoundly influences plant growth and development by participating in the synthesis of amino acids, proteins, nucleic acids, chlorophyll, and various enzymes (Wu et al., 2025). Both nitrogen deficiency and inappropriate forms of nitrogen adversely affect the physiological metabolism of plants (Persyn et al., 2024). Plants primarily absorb inorganic nitrogen in the form of ammonium (NH_4_^+^) and nitrate (NO_3_^^-^^), exhibiting distinct differences in their uptake, transport, storage, and assimilation processes. The preference for different nitrogen forms varies among plant species and environmental conditions, ultimately affecting plant growth (Xiong et al., 2021; Ito et al., 2023). NH_4_^+^ is a major form of nitrogen transport in plants. Absorbed NH_4_^+^ is sequentially converted to glutamine (Gln) and glutamate (Glu) via glutamine synthetase (GS) and glutamate synthase (GOGAT), which serve as key enzymes with high affinity for NH_4_^+^ (Bedell et al., 1994; Fontaine et al., 2012). However, sole NH_4_^+^ supply restricts cation uptake (e.g., K^+^, Ca²^+^, Mg²^+^, Fe²^+^, Zn²^+^), disrupts phytohormone balance, and inhibits energy production and cell division, ultimately suppressing root growth (Di et al., 2023; Liu et al., 2024). NO_3_^-^ assimilation involves reduction to NO_2_^-^ by nitrate reductase (NR), followed by further reduction to NH_4_^+^ by nitrite reductase (NiR). NR is the rate-limiting enzyme in this process (Campbell, 2002; Andrews et al., 2013). NO_3_^-^ promotes lateral root initiation, increases root length and biomass, and serves as a signaling molecule regulating gene expression related to its own transport and assimilation, lateral root development, leaf growth, and flowering time (Kant, 2018).

In the present study, we first screened the optimal total nitrogen concentration and NH_4_^+^:NO_3_^-^ ratio for adventitious root culture. Using physiological and biochemical assays, comparative transcriptomics, and real-time quantitative reverse transcription polymerase chain reaction (qRT-PCR) verification, we investigated the regulatory network linking nitrogen forms to ginsenoside accumulation. This study provides theoretical support for optimizing nitrogen management to improve ginsenoside yields from ginseng adventitious root culture.

## 2 Materials and methods

### 2.1 Plant materials and culture conditions

Adventitious roots of *Panax ginseng* C. A. Mey. were induced and maintained in our laboratory as described previously (Zhang et al., 2026). The roots were cultured in 150 mL Erlenmeyer flasks containing 75 mL of MS liquid medium (pH 5.8) supplemented with 5.0 mg/L indole-3-butyric acid (IBA) and 30 g/L sucrose. Cultures were incubated in darkness at 25 °C on a rotary shaker at 100 rpm for 35 days, and subcultured according to Huang et al.

To investigate the effects of total nitrogen concentration on the growth and metabolism of *P. ginseng* adventitious roots, total-nitrogen gradients of 0, 5, 10, 15, 20, 25, and 30 mmol/L were established. For the NH_4_^+^:NO_3_^-^ ratio experiment, total nitrogen was fixed at 20 mmol/L with ratios of 0:20 (A), 5:15, 10:10 (C), 15:5, and 20:0 (E). A nitrogen-free group (0:0, F) was set as the blank control. All other culture conditions were kept consistent.

Each treatment included multiple parallel flasks. At sampling, adventitious roots from all flasks within the same treatment were pooled and equally split into three aliquots as biological replicates for subsequent analyses. Three subsamples from these pooled tissues were used for transcriptome sequencing.

### 2.2 Equations

#### 2.2.1 Root fresh weight and dry weight

Fresh weight (FW) was determined by rinsing adventitious roots with tap water on a metal sieve, followed by blotting surface moisture with filter paper. Dry weight (DW) was measured after drying the roots at 55 °C for 48 h until constant weight.

#### 2.2.2 Quantification of auxin and cytokinin contents

Frozen samples ground to a fine powder in liquid nitrogen were mixed with an appropriate volume of phosphate-buffered saline (PBS), vortexed for 5 min, and centrifuged at 12,000 × *g* for 10 min. The resulting supernatant was collected as the sample extract. Auxin (IAA) and cytokinin (CTK) levels were determined using a plant hormone enzyme-linked immunosorbent assay (ELISA) kit (Shanghai Enzyme-linked Biotechnology Co., Ltd., China) following the manufacturer’s protocol. This immunoassay method was adopted for comparative quantification to evaluate relative changes in hormone abundance across different nitrogen treatments.

#### 2.2.3 Measurement of nitric oxide content

NO content was determined using a visible spectrophotometric method with a NO assay kit (Shanghai Enzyme-linked Biotechnology Co., Ltd., China). The standard curve regression equation was $Y=0.008X-0.0103(R^{2}=0.9986)$. NO content was calculated based on fresh weight as follows; where ΔA is the absorbance change, V_total_ is the total reaction volume (0.2 mL), V_sample_ is the sample volume (0.01 mL), and W is the sample fresh weight (g).


$$NO\;content(\mu mol\cdot g^{-1})=\frac{\left(\Delta A+0.0103\right)}{0.008}\times \frac{V_{total}}{V_{sample}\times W}\times 10^{-3}$$

#### 2.2.4 Assay of antioxidant enzyme activities

Activities of peroxidase (POD), catalase (CAT), and superoxide dismutase (SOD) were measured using commercial kits (Shanghai Enzyme-linked Biotechnology Co., Ltd., China).where ΔA is the absorbance change, V_total_ is the total reaction volume (0.2 mL), V_sample_ is the sample volume (0.01 mL), W is the sample fresh weight (g), T is the reaction time (1 min), ϵ is the molar extinction coefficient (L·mol^-^¹·cm^-^¹), and d is the optical path length (cm).


$$POD=\frac{\Delta A\times V_{total}}{W\times V_{sample}\times 0.005\times T}$$

$$CAT=\frac{\Delta A\times V_{total}\times 10^{6}}{\epsilon \times d\times W\times V_{sample}\times T}$$

$$SOD=\frac{Inhibition\;Percentage\times V_{total}}{(1-Inhibition\;Percentage)\times W\times V_{sample}}$$

#### 2.2.5 Experimental verification of NO effects on ginsenoside accumulation

Ginseng adventitious roots were treated with exogenous NO donor sodium nitroprusside (SNP) and NO scavenger 2-(4-carboxyphenyl)-4,4,5,5-tetramethylimidazoline-1-oxyl-3-oxide (cPTIO). Four experimental treatments were set as follows (Zhan et al., 2014): Control (MS) (Tao et al., 2023); 20 mM KNO_3_; (3) 20 mM KNO_3_^+^ 100 µM cPTIO; (4) 20 mM KNO_3_^+^ 100 µM cPTIO ^+^ 100 µM SNP. Each treatment contained three biological replicates, and samples were harvested after 35 d of culture. One portion of samples was weighed and dried for ginsenoside content analysis; the remaining samples were snap-frozen in liquid nitrogen and stored at −80 °C for subsequent measurements.

Adventitious roots were cultured in medium containing 20 mM KNO_3_, supplemented with SNP at concentrations of 0, 25, 50, 100, 150, and 200 µM. SNP solutions were filtered through a 0.22 μm filter membrane before addition to the medium. Samples were harvested after 35 d of cultivation. Other culture conditions were consistent with section 2.1. Samples were weighed and dried for ginsenoside determination, and all experiments were carried out in triplicate.

### 2.3 Ginsenoside extraction and analysis

Dried adventitious roots were ground into powder and passed through a 60-mesh sieve. A 0.3 g aliquot of the powder was placed in a round-bottom flask and extracted with 70 mL of methanol by refluxing at 75 °C for 3 h. After cooling, the supernatant was collected and evaporated to dryness at 60 °C using a rotary evaporator. The residue was dissolved in 5 mL of methanol, followed by the addition of 25 mL of ether. After vortexing for 45 s, the mixture was transferred to a separatory funnel. Following 40 min of phase separation, the lower saponin-containing phase was collected. The saponins were then extracted four times with water-saturated n-butanol, each time centrifuged at 12,000 rpm for 5 min. The butanol layers were pooled and evaporated to dryness via rotary evaporation. The final residue was dissolved in chromatographic-grade methanol (5 mL per 0.3 g sample) and filtered through a 0.45 μm membrane prior to HPLC analysis.

Ginsenoside analysis was performed using high-performance liquid chromatography (HPLC) following the method described by Lin (Lin et al., 2025). Chromatographic separation was conducted on a reversed-phase C18 column (Acchrom, 5 μm, 4.6 × 250 mm) with the column temperature maintained at 25 ± 0.5 °C. The mobile phase consisted of ultrapure water (A) and HPLC-grade acetonitrile (B), delivered at a flow rate of 1.0 mL·min^-^¹ using a binary gradient elution program as follows: 0–13 min, 77% A/23% B; 13–33 min, 54% A/46% B; 33–45 min, 32% A/68% B; 45–60 min, 0% A/100% B; 60–66 min, 77% A/23% B. The injection volume was 10 μL, and detection was carried out using a UV detector at a wavelength of 203 nm.

Mixed ginsenoside standard stock solution was prepared as follows: 1 mg of each of seven major ginsenoside standards (Rg1, Re, Rf, Rb1, Rc, Rb2, Rd) was accurately weighed, quantitatively transferred into a 2-mL brown chromatographic vial and dissolved in 1 mL of HPLC-grade methanol to obtain mixed stock solution with each ginsenoside at 1 mg·mL^-^¹. Standard curves were established for the seven ginsenoside monomers. Working standard solutions were prepared at the following concentrations: 0.01 mg·mL^-^¹, 0.15 mg·mL^-^¹, 0.2 mg·mL^-^¹, 0.25 mg·mL^-^¹, 0.3 mg·mL^-^¹, and 0.35 mg·mL^-^¹. Each standard solution was analyzed by HPLC with three technical replicates. The retention times of reference standards were Re (10.12 min), Rg1 (12.55 min), Rf (26.27 min), Rb1 (27.20 min), Rc (28.52 min), Rb2 (29.69 min), and Rd (31.73 min). Standard curves for the seven ginsenosides were plotted using standard concentration (X) as the abscissa and chromatographic peak area (Y) as the ordinate. The obtained standard-curve parameters are shown in **Supplementary Table S8**.

### 2.4 Transcriptome sequencing and data analysis

RNA library construction and Illumina sequencing were performed by Biomarker Technologies Co., Ltd. (Beijing, China). RNA concentration and purity were measured using a NanoDrop 2000 spectrophotometer (Thermo Fisher Scientific, USA). RNA integrity was assessed using the RNA Nano 6000 Assay Kit on an Agilent Bioanalyzer 2100 system (Agilent Technologies, USA). One microgram of total RNA from each sample was used for library construction. Sequencing libraries were generated using the Hieff NGS Ultima Dual-mode mRNA Library Prep Kit for Illumina (Yeasen Biotechnology (Shanghai) Co., Ltd.) following the manufacturer’s protocols, and index codes were added to distinguish different samples.

Raw FASTQ reads were processed using Perl scripts for strict quality filtering to guarantee the reliability of downstream analyses. Reads were filtered according to the following criteria (Zhan et al., 2014): remove reads containing adapter sequences; (2) discard reads in which more than 40 % of bases possessed a Q-value < 15, remove trailing poly-G sequences, and eliminate reads with sequence complexity lower than 10 %; (3) remove reads shorter than 100 bp; (4) discard reads with more than five ambiguous N bases in single-end reads. After filtering, high-quality clean data in FASTQ format were obtained (**Supplementary Table S5**). Q20, Q30, GC content and duplication level were calculated for quality-control evaluation. All subsequent analyses relied on these high-quality clean reads. Clean reads were mapped to the *P. ginseng* IPGA v1.1 reference genome (http://ginsengdb.snu.ac.kr/ total predicted genes: 59,352) using Hisat2 v2.0.4 (Kim et al., 2015). Nature methods, 2015, 12(4): 357-360. Mapping rates ranged from 94.8% to 96.7% (**Supplementary Table S6**). Gene-level expression abundances were estimated using StringTie with a maximum-flow algorithm. FPKM (Fragments Per Kilobase of transcript per Million fragments mapped) was applied to normalize read counts and quantify transcript-/gene-level expression values. In this formula, cDNA Fragments represents the number of fragments (paired-end reads) uniquely mapped to a given transcript; Mapped Fragments (Millions) denotes the total number of mapped fragments in millions; Transcript Length (kb) is the transcript length in kilobases (10³ bases).


$$FPKM=\frac{\text{cDNAFragments}}{Mapped\;Fragments\;(Millions)*Transcript\;Length(kb)}$$

Differential expression analysis was performed jointly using DESeq2 (v1.30.1, test = “Wald”, fitType = “parametric”) and edgeR (v3.32.1, dispersion = 0.01, agricultural-setting) based on three biological replicates./Differentially expressed genes (DEGs)represented the intersection of two analytical outputs; genes with FDR < 0.01 and fold change (FC) ≥ 2 in both software were identified as DEGs. For heat-map visualization, FPKM values of DEGs were normalized by Z-score transformation. All raw transcriptome sequencing data have been deposited in the NCBI Sequence Read Archive (SRA) under accession PRJNA1484283.

### 2.5 Functional annotation of DEGs

Gene Ontology (GO) enrichment analysis of DEGs was implemented with the clusterProfiler R package based on the Wallenius non-central hypergeometric distribution. For pathway enrichment, the Kyoto Encyclopedia of Genes and Genomes (KEGG) database was adopted. KOBAS combined with clusterProfiler was used to test the statistical enrichment of DEGs in KEGG metabolic pathways.

### 2.6 Quantitative real-time PCR validation

#### 2.6.1 Total RNA extraction

Total RNA was extracted from ginseng adventitious roots using the Total RNApure Reagent Kit (Beijing Zhuangmeng International Biological Gene Technology Co., Ltd.). Pre-chilled mortars and pestles were used to grind 0.5 g of frozen sample in liquid nitrogen to a fine powder. The powder was mixed with 5 mL of Total RNApure Reagent and centrifuged at 13,400 × *g* for 10 min at 4 °C. The supernatant was transferred to a new tube, mixed with 1 mL of chloroform, vortexed thoroughly, and centrifuged at 13,400 × *g* for 15 min at 4 °C. The aqueous phase was collected, mixed with an equal volume of isopropanol, and incubated at room temperature for 5 min. After centrifugation at 13,400 × *g* for 10 min at 4 °C, the supernatant was discarded. The pellet was washed with 5 mL of 75% ethanol (diluted with DEPC-treated water), vortexed vigorously, and centrifuged at 9,391 × *g* for 5 min at 4 °C. The supernatant was removed, and the pellet was air-dried for 5 min at room temperature. The RNA was dissolved in 30 μL of DEPC-treated water and stored at −80 °C. RNA concentration and purity were assessed by absorbance at 260 nm and 280 nm. RNA integrity was evaluated on a 1% agarose gel.

#### 2.6.2 cDNA synthesis

Total RNA was reverse-transcribed into cDNA using the Zhuangmeng Biotechnology Reverse Transcription Kit according to the manufacturer’s protocol. The resulting cDNA was stored at −20 °C.

#### 2.6.3 Primer design and qRT-PCR

To verify the reliability of RNA-seq data, twelve representative DEGs with distinct expression patterns were selected for qRT-PCR analysis. Gene-specific primers for qRT-PCR were designed using Primer Premier 5.1 software (**Supplementary Table S7**). The 18S rRNA gene was used as the internal control. qRT-PCR was performed on a Bio-RAD real-time PCR system in a 10 μL reaction volume. The PCR program was as follows: 95 °C for 10 min (pre-denaturation), followed by 40 cycles of 95 °C for 10 s, 60 °C for 10 s, and 72 °C for 20 s. Relative gene expression levels were calculated using the 2^-^ΔΔCT method, normalized to the internal control.

### 2.7 Statistical analysis

Data of physiological indicators were expressed as mean ± standard deviation (SD). Partial raw data are listed in **Supplementary Table S9**. The significant difference between the two groups’ mean values was evaluated using a *t*-test (*P* < 0.05). The significant differences among multiple groups were assessed using analysis of variance (ANOVA) (*P* < 0.05), supplemented by Duncan’s multiple-range test for *post hoc* pairwise comparisons (*P* < 0.05). For transcriptomic data, DEGs were identified by joint analysis of DESeq2 and edgeR. Genes with FDR < 0.01 and FC ≥ 2 were considered significantly differentially expressed.

## 3 Results

### 3.1 Growth and ginsenoside accumulation of *Panax ginseng* adventitious roots: screening of optimal nitrogen concentration and responses to various NH_4_^+^:NO_3_^-^ ratios

There was a strong response of adventitious root biomass and ginsenoside accumulation to the nitrogen supply (**Figure 1A**). When the nitrogen concentration increased from 0 to 20 mmol·L^−1^, the fresh weight, dry weight, and total ginsenoside content continued to increase. When the concentration exceeded 20 mmol·L^−1^, the growth and saponin synthesis were not further enhanced. The 20 mmol·L^−1^ treatment was determined as appropriate to conduct the subsequent ammonium nitrate ratio tests.

**Figure 1:**
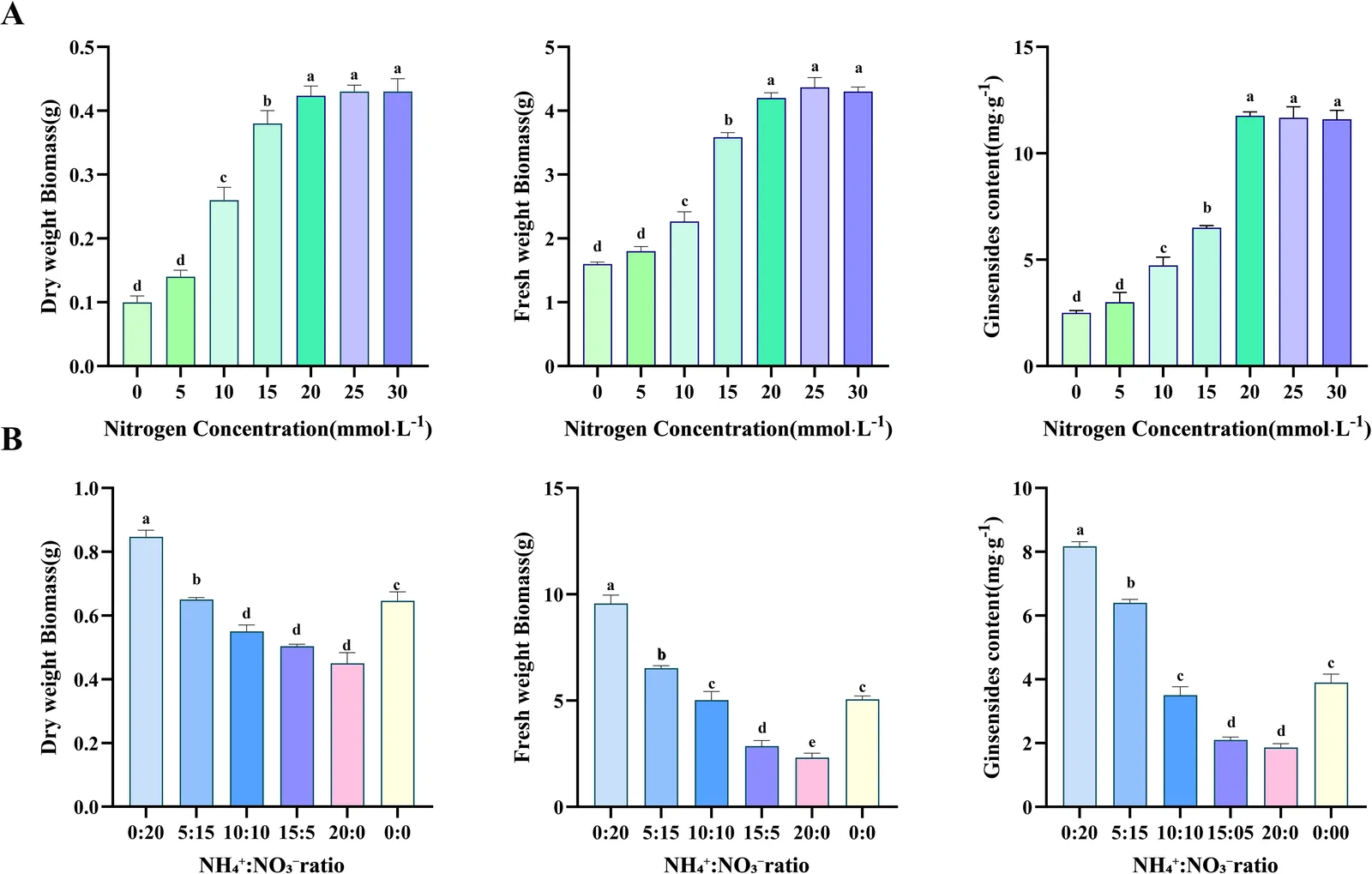
Biomass and total ginsenoside content of *Panax ginseng* adventitious roots under sequential nitrogen treatments. **(A)** Effects of gradient nitrate concentrations (0–30 mmol L^-^¹) on dry weight, fresh weight, and total ginsenoside concentration for optimum nitrogen concentration screening. **(B)** Phenotypic responses to different NH_4_^+^:NO_3_^-^ ratios under a fixed total nitrogen concentration of 20 mmol L^-^¹. Different lowercase letters indicate significant differences among treatments (*P* < 0.05).

The phenotype of different ammonium nitrate ratio treatment groups was markedly different at 20 mmol·L^−1^ nitrogen (**Figure 1B**). The dry weight, fresh weight, and total ginsenoside content of adventitious roots were the highest under the single nitrate-nitrogen treatment (0:20). As the proportion of ammonium-nitrogen increased, adventitious root growth and ginsenoside synthesis were inhibited. Biomass and saponin accumulation were the lowest in the pure ammonium-nitrogen treatment (20:0).

### 3.2 Comparative results of ginseng adventitious root sequencing

To determine the molecular basis underlying growth and ginsenoside accumulation phenotypes induced by different nitrogen forms, transcriptome sequencing was conducted on nine adventitious root samples with three nitrogen treatments: sole nitrate (A, NH_4_^+^:NO_3_^-^ = 0:20), sole ammonium (E, NH_4_^+^:NO_3_^-^ = 20:0), and nitrogen-free control (F, NH_4_^+^:NO_3_^-^ = 0:0). The Pearson correlation heat map indicates the repeated biological correlation of the overall transcriptional profiles between the samples (**Figure 2A**). Principal-component analysis (PCA) was also conducted to evaluate the overall similarity and divergence among all treatment samples, with the results presented in (**Supplementary Figure S2**). The screening thresholds of false discovery rate (FDR) < 0.01 and difference multiple ≥ 2 were used. The Venn diagram showed three groups of unique and common differential genes (**Figure 2B**). The volcanic map indicates the overall distribution of up- and down-regulated differential genes in each group (**Figure 2C**). There were 2913 DEGs (1314 up-regulated and 1599 down-regulated) in E vs. A, 1745 DEGs (632 up-regulated and 1113 down-regulated) in F vs. A, and 1308 DEGs in F vs. E (667 upregulated and 641 downregulated).

**Figure 2:**
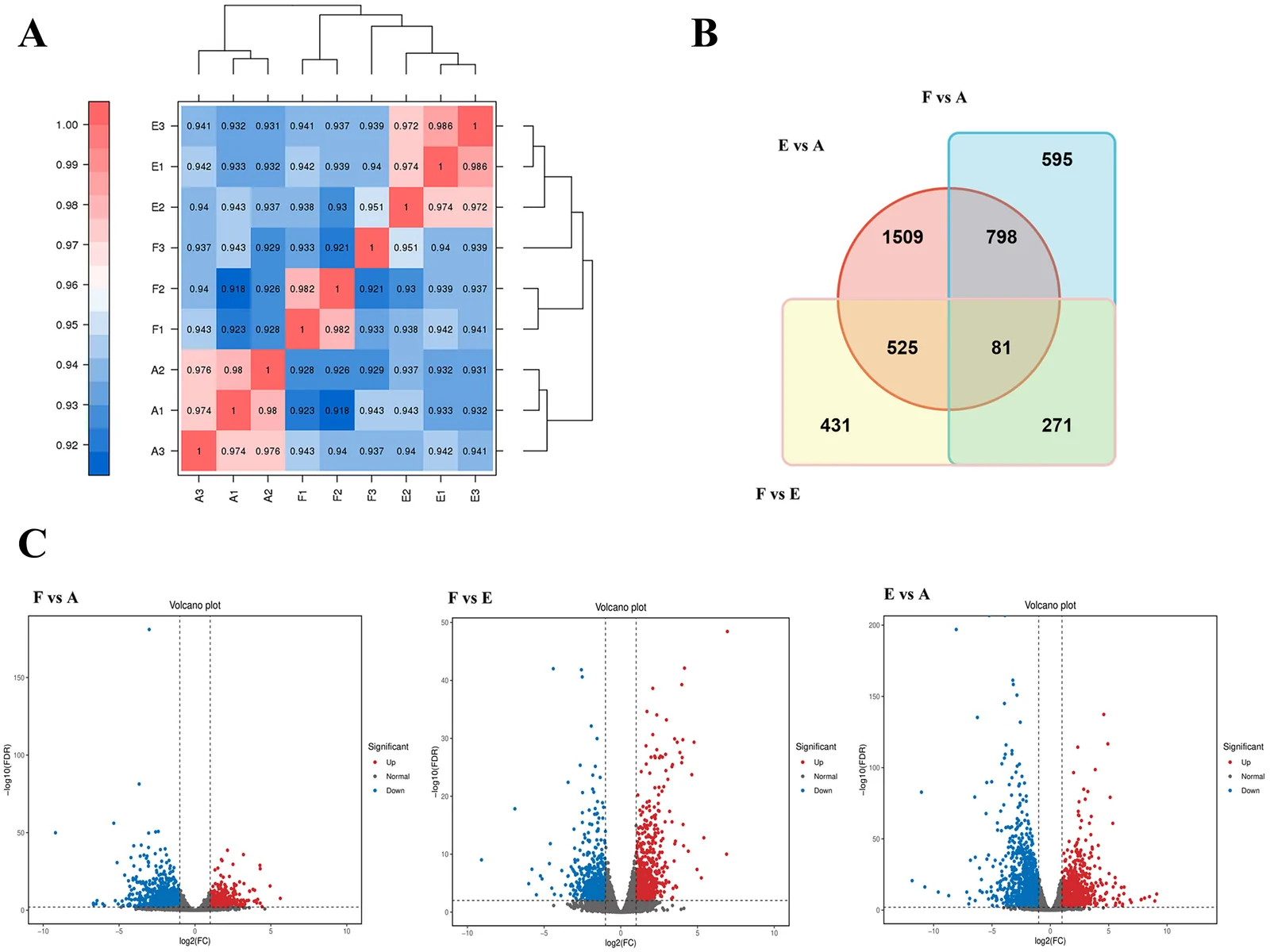
Global transcriptomic characteristics of *Panax ginseng* adventitious roots under three nitrogen treatments. **(A)** Pearson correlation heatmap of gene expression among all nine biological samples. The color gradient represents pairwise correlation coefficients. Hierarchical clustering was performed based on normalized gene expression profiles. **(B)** Venn diagram illustrating the common and unique DEGs among comparison groups. **(C)** Volcano plots showing DEG distribution in each comparison group. DEGs were screened jointly by DESeq2 and edgeR with thresholds: FDR<0.01 and fold change≥2. (A: sole nitrate; E: sole ammonium; F: nitrogen-free control).

### 3.3 Functional enrichment analysis of DEGs indicates key pathways responsive to different nitrogen forms

The KEGG enrichment bubble diagram was used to analyze the pathway changes in up and down-regulated differential genes under the pairwise comparison of the three groups (**Figures 3A, B**). For up-regulated DEGs, in the comparison of F vs. A and F vs. E groups, nitrogen deficiency treatment was associated with the enrichment of up-regulated DEGs in plant hormone signaling, MAPK signaling pathway, nitrogen metabolism, phenylpropanoid biosynthesis, and other stress and secondary metabolism-related pathways. In the comparison of E vs. A (ammonium-nitrogen vs nitrate-nitrogen), the number of DEGs in plant hormone signal transduction was the largest, and the MAPK signaling pathway, zeatin biosynthesis, sesquiterpene and triterpene biosynthesis pathways were enriched. Hence, the two forms of nitrogen, ammonium and nitrate, induced marked transcriptional differentiation of hormone signals and terpenoid metabolism. For downregulated differential genes, F vs. A and F vs. E suggested that nitrogen deficiency may suppress endoplasmic reticulum protein processing, hormone signaling, carotenoid synthesis, and other pathways to maintain normal growth, whereas the number of downregulated genes enriched in the phenylpropanoid biosynthesis pathway was the highest in E vs. A, accompanied by marked changes in glutathione and nitrogen metabolism, and in MAPK and hormone signaling pathways, suggesting that nitrate-nitrogen could maintain higher transcriptional activity of primary metabolism, antioxidant systems, and secondary metabolism pathways compared to ammonium nitrogen.

**Figure 3:**
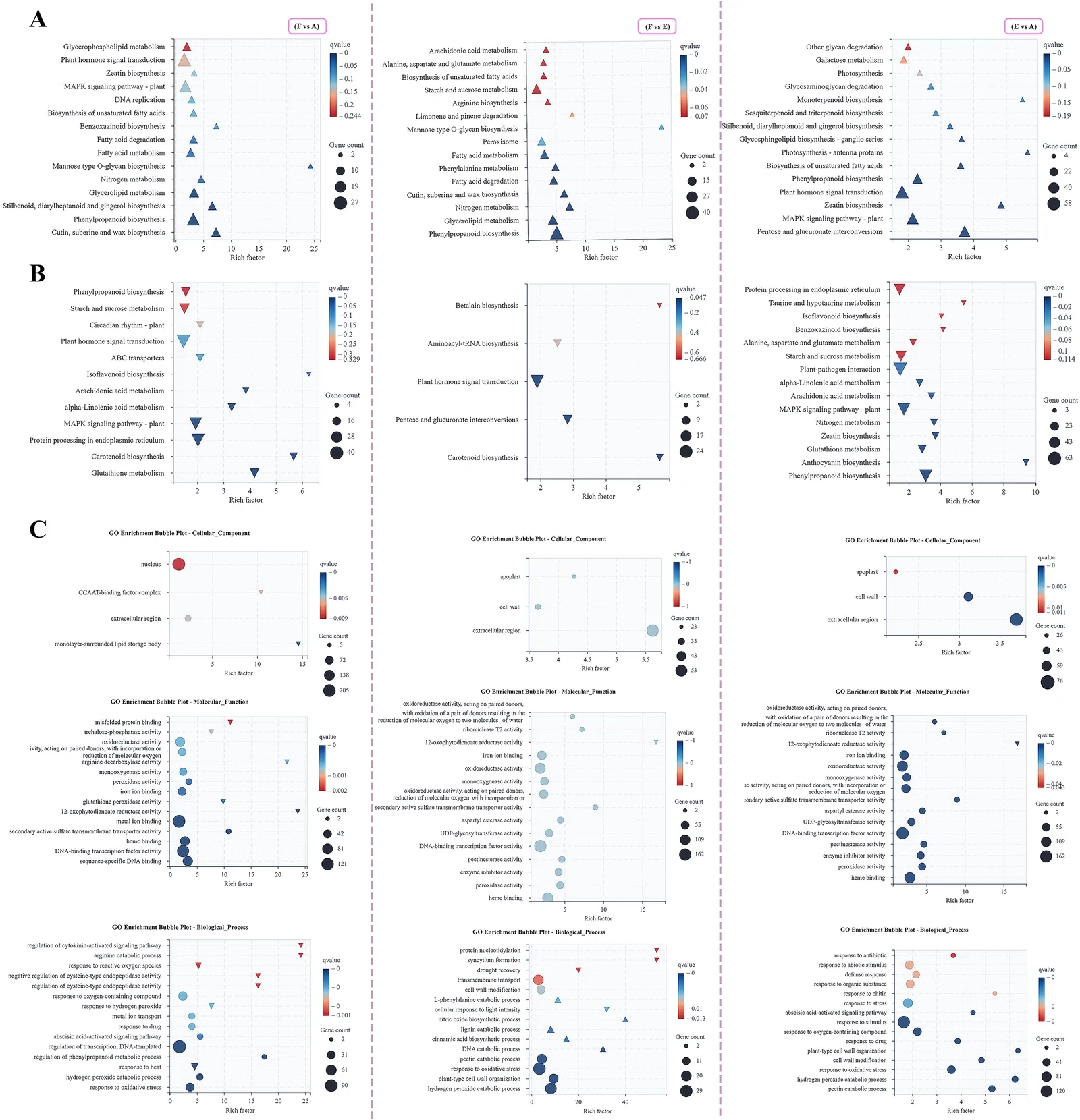
Enrichment analysis of DEGs. **(A)** KEGG bubble plot for up-regulated DEGs; **(B)** KEGG bubble plot for down-regulated DEGs; **(C)** GO enrichment. Terms/pathways with adjusted P-value < 0.05 are shown. (A: sole nitrate; E: sole ammonium; F: nitrogen-free control).

GO analysis was conducted on the DEGs in the three groups of pairwise comparisons. The overall distribution of the DEGs in the three first-level GO classifications of biological processes (BP), cellular components (CC), and molecular functions (MF) is shown in **Supplementary Figure S1**. The GO enrichment bubble diagram indicates the global functional changes (**Figure 3C**). At the cell component level, F vs. A DEGs were mainly enriched in the nucleus and extracellular region; the DEGs in the F vs. E and E vs. A were markedly enriched in the apoplast, cell wall, and extracellular region.

At the molecular functional level, GO terms related to oxidoreductase activity, ion binding, transferase activity, and DNA-binding transcription factor activity were significantly enriched in the three groups; a large number of redox-related items were markedly enriched in E vs. A. At the biological process level, GO terms related to hormone signal regulation, stress response, and transcriptional regulation were enriched in F vs. A; GO terms related to protein modification, cell wall remodeling, and oxidative stress response were enriched in F vs. E; and DEGs in E vs. A were enriched in defense response, oxidative stress, hydrogen peroxide decomposition, and secondary metabolic biosynthesis.

### 3.4 Expression patterns of core genes involved in ginsenoside biosynthetic pathway

Given the significant enrichment of the terpenoid biosynthesis pathway in the functional enrichment analysis, we further analyzed the transcriptional changes of key structural genes involved in the ginsenoside biosynthesis pathway (**Figure 4**; **Supplementary Table S1**). A total of 11 genes (corresponding to HMGR, ispH, GPS, FPS, SS, SQE, DDS, and β-AS, among others) showed markedly different expression levels across nitrogen treatments. The heatmap revealed that most of these DEGs exhibited higher transcript levels under nitrate treatment (A) than under other conditions (**Figure 4**), whereas ammonium treatment (E) suppressed the transcription of most saponin biosynthesis-related genes. This transcriptional pattern is consistent with the phenotypic observations, as nitrate treatment led to both upregulation of ginsenoside biosynthesis genes and increased ginsenoside accumulation, while ammonium treatment showed the opposite effects.

**Figure 4:**
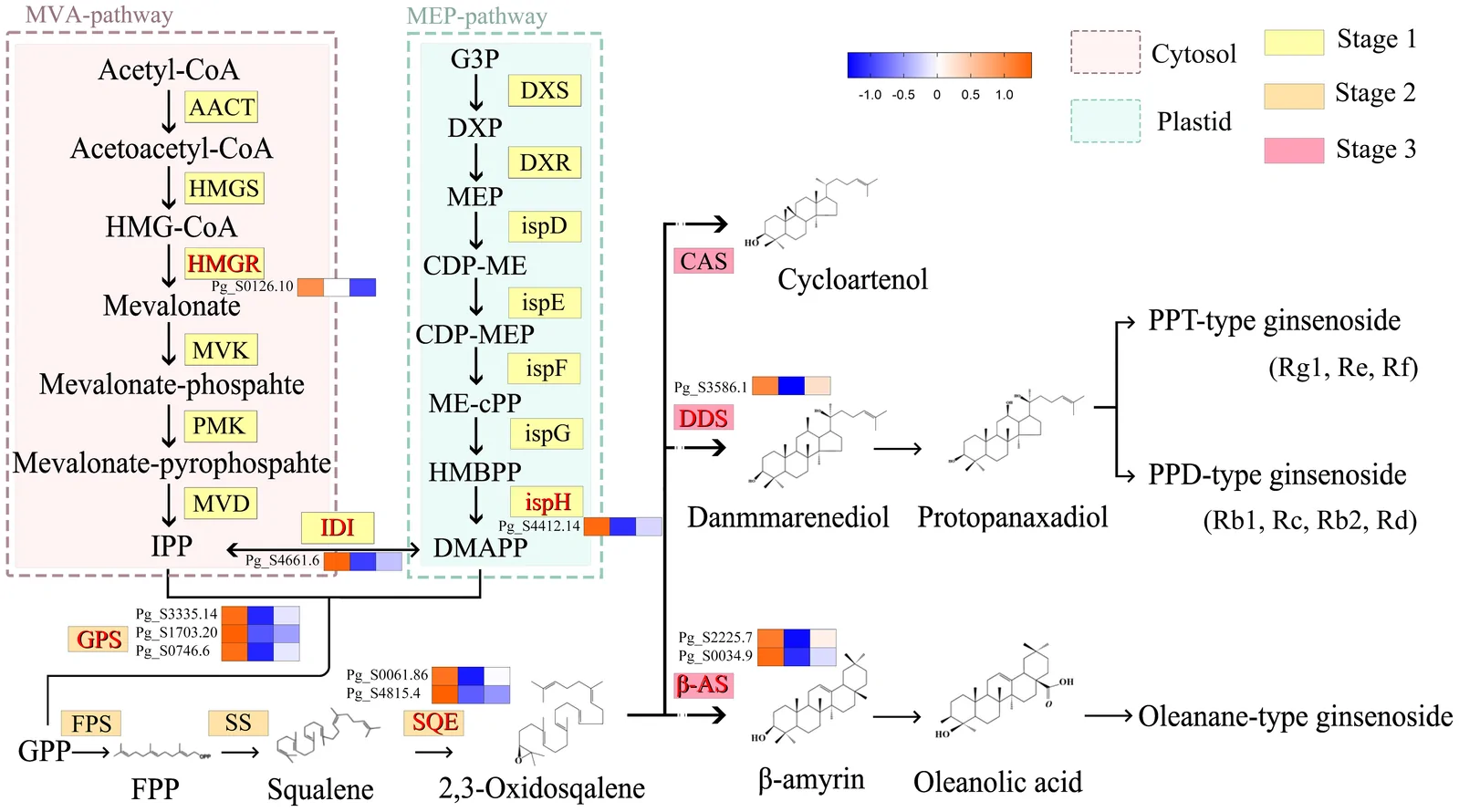
Schematic diagram of the ginsenoside biosynthesis pathway and expression profiles of key enzyme-coding genes. The color bar represents standardized gene expression based on FPKM values, corresponding to A, E, and F (from left to right). A: sole nitrate; E: sole ammonium; F: nitrogen-free control.

### 3.5 Differential expression of DEGs associated with plant hormone pathways and quantification of endogenous phytohormone contents under different nitrogen treatments

Plant hormones are core upstream regulatory factors that connect nutrient signals and regulate plant growth and secondary metabolism. The transcriptional changes in the IAA, CTK, ethylene, jasmonic acid, gibberellin (GA), and abscisic acid (ABA) pathways were analyzed. Several key genes involved in hormone synthesis and signaling pathways showed substantial nitrogen form-dependent transcriptional responses (**Figures 5A, B**; **Supplementary Table S2**). Most of the DEGs were in the IAA and CTK pathways, and under different treatment conditions, most had higher expression levels in the nitrate-nitrogen treatment group, whereas their expression levels under the ammonium-nitrogen treatment were the lowest. Unlike the IAA and CTK pathways, the transcriptional pattern of GA pathway-related genes was the opposite, and their expression levels increased in the ammonium-nitrogen (E) and nitrogen-free (F) groups.

**Figure 5:**
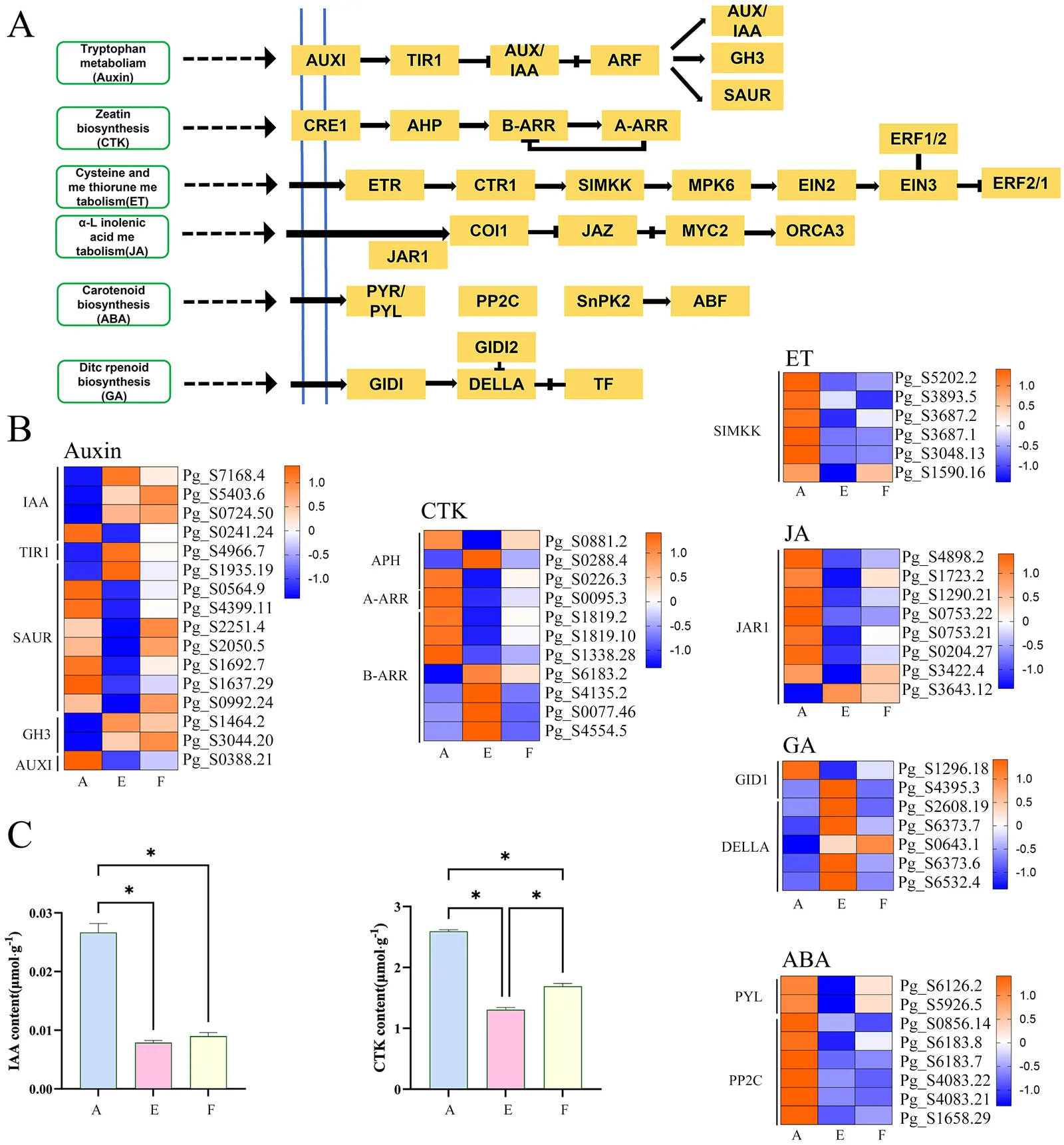
Analysis of plant hormone signaling pathways and quantification of endogenous hormone contents under different nitrogen treatments. **(A)** Schematic diagram of plant hormone signal transduction. **(B)** Heatmap showing the expression profiles of key genes involved in six plant hormone signaling pathways. **(C)** Determination of endogenous IAA and CTK concentrations in ginseng adventitious roots. Asterisks indicate significant differences (*P* < 0.05). A: sole nitrate treatment; E: sole ammonium treatment; F: nitrogen-free control.

Consistent with the transcription of hormone signaling pathway genes, the quantitative results of endogenous hormones indicated marked differences in the accumulation levels of IAA and CTK between the different treatments (**Figure 5C**). The content of endogenous IAA in adventitious roots of the nitrate-nitrogen treatment (A) was substantially higher than that in the ammonium-nitrogen treatment (E) and the nitrogen-free control group (F). The content of CTK peaked in group A. Single ammonium-nitrogen supply substantially inhibited the accumulation of IAA and CTK, and the hormone content of the nitrogen-free treatment (F) was markedly higher than that of the E group.

### 3.6 Antioxidant enzyme activities and antioxidant-related DEGs under different nitrogen forms

The activity of core antioxidant enzymes and the expression levels of the corresponding genes were investigated. The results of the enzyme activity determination showed that there were marked differences among the groups (**Figure 6A**; **Supplementary Table S3**). SOD activity was the highest in the nitrate-nitrogen group (A), followed by the ammonium-nitrogen group (E), and was lowest in the nitrogen-free group (F). The activities of CAT and POD peaked under the single ammonium-nitrogen treatment (E), and the activities of the two enzymes in the nitrogen-free group decreased markedly. These results show that ammonium-nitrogen treatment was associated with higher CAT and POD activities, whereas nitrate-nitrogen treatment was associated with higher SOD activity. The results of the transcriptome heat map indicated that many SOD-coding genes had higher expression levels in group A; most CAT and POD genes were upregulated under ammonium-nitrogen treatment (E) (**Figure 6B**).

**Figure 6:**
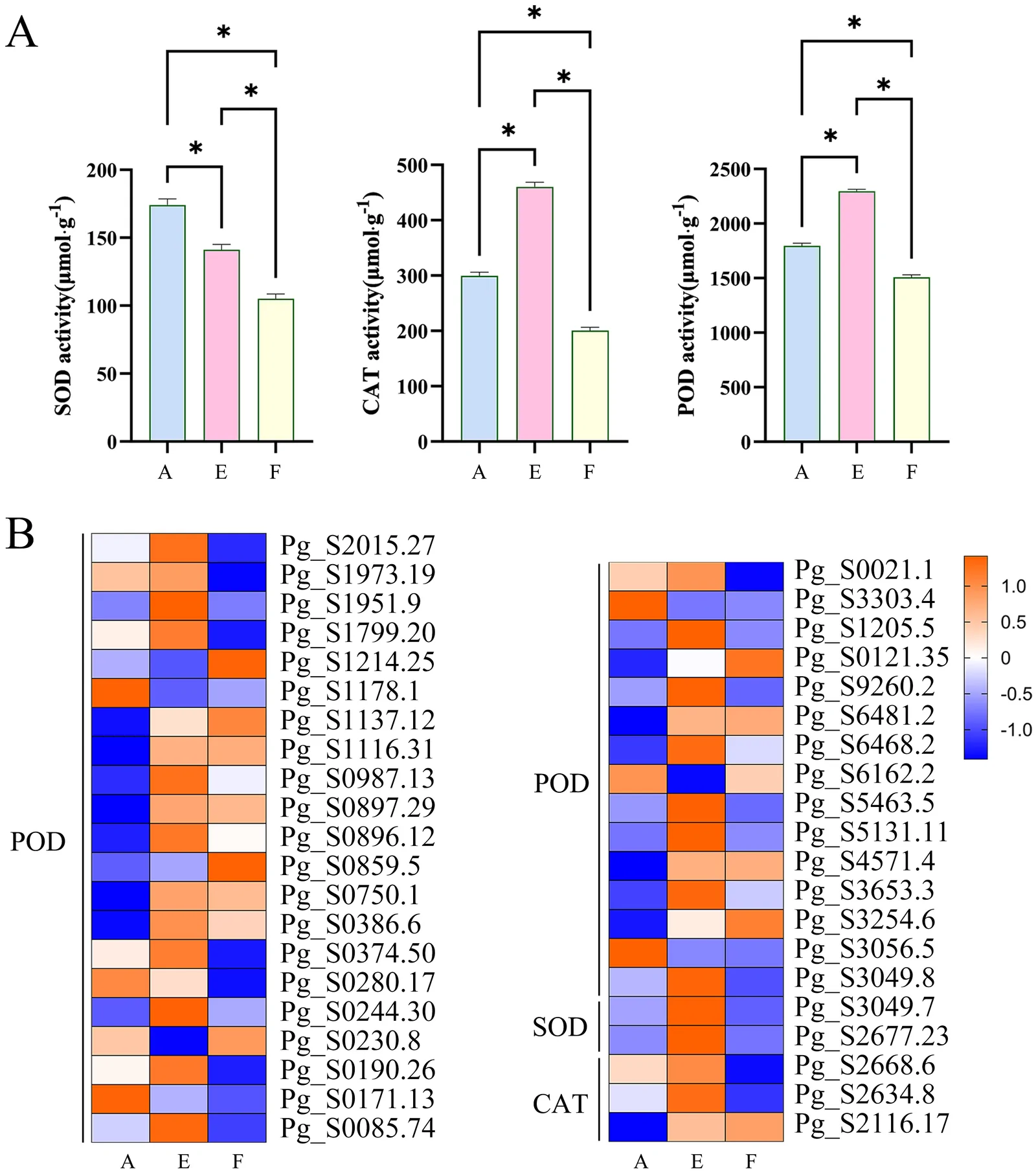
Physiological and transcriptomic characteristics of the antioxidant system in *Panax ginseng* adventitious roots under three nitrogen treatments. **(A)** Enzymatic activities of SOD, CAT and POD. Asterisks denote significant differences between treatments (*P* < 0.05). **(B)** Expression heatmaps of genes encoding SOD, CAT and POD based on FPKM values. The color scale indicates normalized gene expression (red: high expression; blue: low expression). A: sole nitrate; E: sole ammonium; F: nitrogen-free control.

### 3.7 Nitrogen metabolism genes, endogenous NO, and NO function in regulating growth and ginsenoside accumulation of ginseng adventitious roots

The expression levels of key genes involved in nitrogen assimilation were analyzed (**Figure 7A**; **Supplementary Table S4**). Genes encoding the nitrate transporter, *NRT*, nitrate reductase (*NR*), and nitrite reductase (*NiR*) exhibited higher transcript abundance under nitrate treatment (A), whereas the ammonium transporter gene, *AMT*, showed higher transcript abundance under ammonium treatment (E). Endogenous NO concentration was substantially higher in nitrate-treated roots than in the ammonium-treated and nitrogen-free controls (F), both of which greatly suppressed NO accumulation (**Figure 7B**).

**Figure 7:**
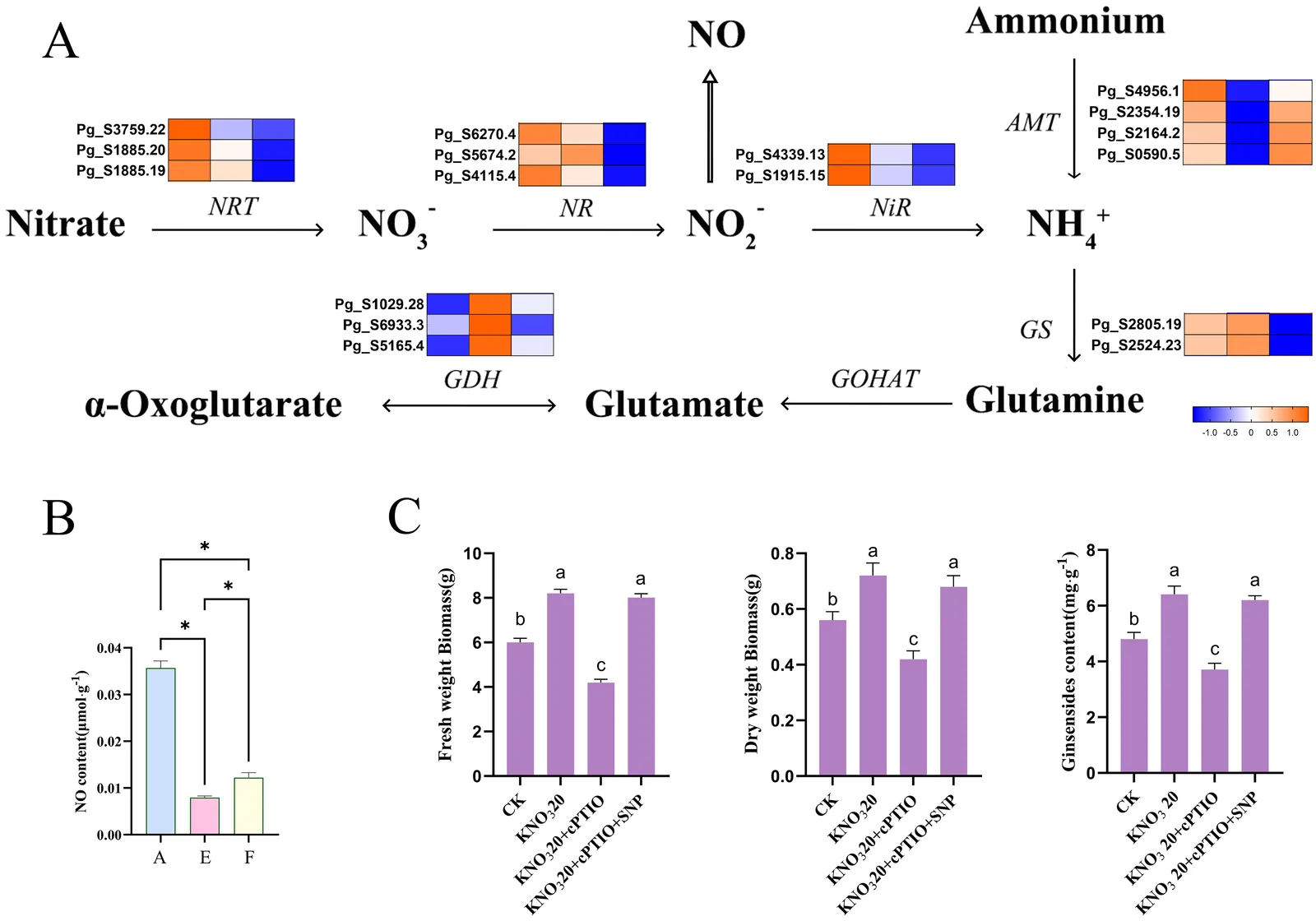
Nitrogen metabolism pathway analysis, NO determination, and functional verification of NO in ginseng adventitious roots. **(A)** Heatmap analysis of differentially expressed genes involved in the nitrogen metabolism pathway; **(B)** Endogenous NO concentration in ginseng adventitious roots under three nitrogen treatments. Asterisks indicate significant differences (*P* < 0.05); **(C)** Fresh weight, dry weight, and total ginsenoside content under nitrate treatment combined with the NO scavenger cPTIO and NO donor SNP. CK: control group; KNO_3_: nitrate treatment group; KNO_3_^+^ cPTIO: nitrate plus NO scavenger treatment group; KNO_3_^+^ cPTIO^+^ SNP: nitrate, cPTIO and SNP combined treatment group. Different lowercase letters indicate significant differences (*P* < 0.05). A: sole nitrate; E: sole ammonium; F: nitrogen-free control; CK: standard MS medium.

To investigate whether NO signaling was involved in nitrate-induced growth and ginsenoside accumulation, the NO scavenger cPTIO and the exogenous NO donor SNP were applied (**Figure 7C**). KNO_3_ treatment promoted the growth of adventitious roots considerably, and the fresh and dry weights increased by 16.6% and 29.0%, respectively, compared to the control group. After applying cPTIO under nitrate-nitrogen conditions, the fresh and dry weights of adventitious roots decreased by 26.5% and 38.2%, respectively, compared to the control. Subsequent SNP supplementation restored root biomass to levels substantially higher than those of the cPTIO treatment and the control groups.

The pattern of ginsenoside accumulation was consistent with the change in biomass: KNO_3_ treatment increased ginsenoside content by 33%. cPTIO application decreased ginsenoside content by 42.2% and ginsenoside yield by 40.7%. Subsequent SNP supplementation increased ginsenoside content to 120.3% of the control and ginsenoside yield to 1.47 fold of the control. Both biomass and ginsenoside content under SNP supplementation were substantially higher than those under cPTIO treatment alone.

### 3.8 Validation of transcriptome reliability using qRT-PCR results

To validate the reliability of the transcriptomic data, 12 DEGs with marked changes in expression levels were selected for qRT-PCR validation. The expression levels of genes (*Pg_S4661.6, Pg_S0061.86, Pg_S4815.4, Pg_S1464.2, Pg_S2225.7, Pg_S3586.1, Pg_S0724.50, Pg_S0897.29, Pg_S1029.28, Pg_S2524.23, Pg_S0590.5*, and *Pg_S1915.15*) exhibited high consistency between qRT-PCR and RNA-sequencing results, demonstrating the robustness and reliability of the transcriptomic analysis (**Figure 8**).

**Figure 8:**
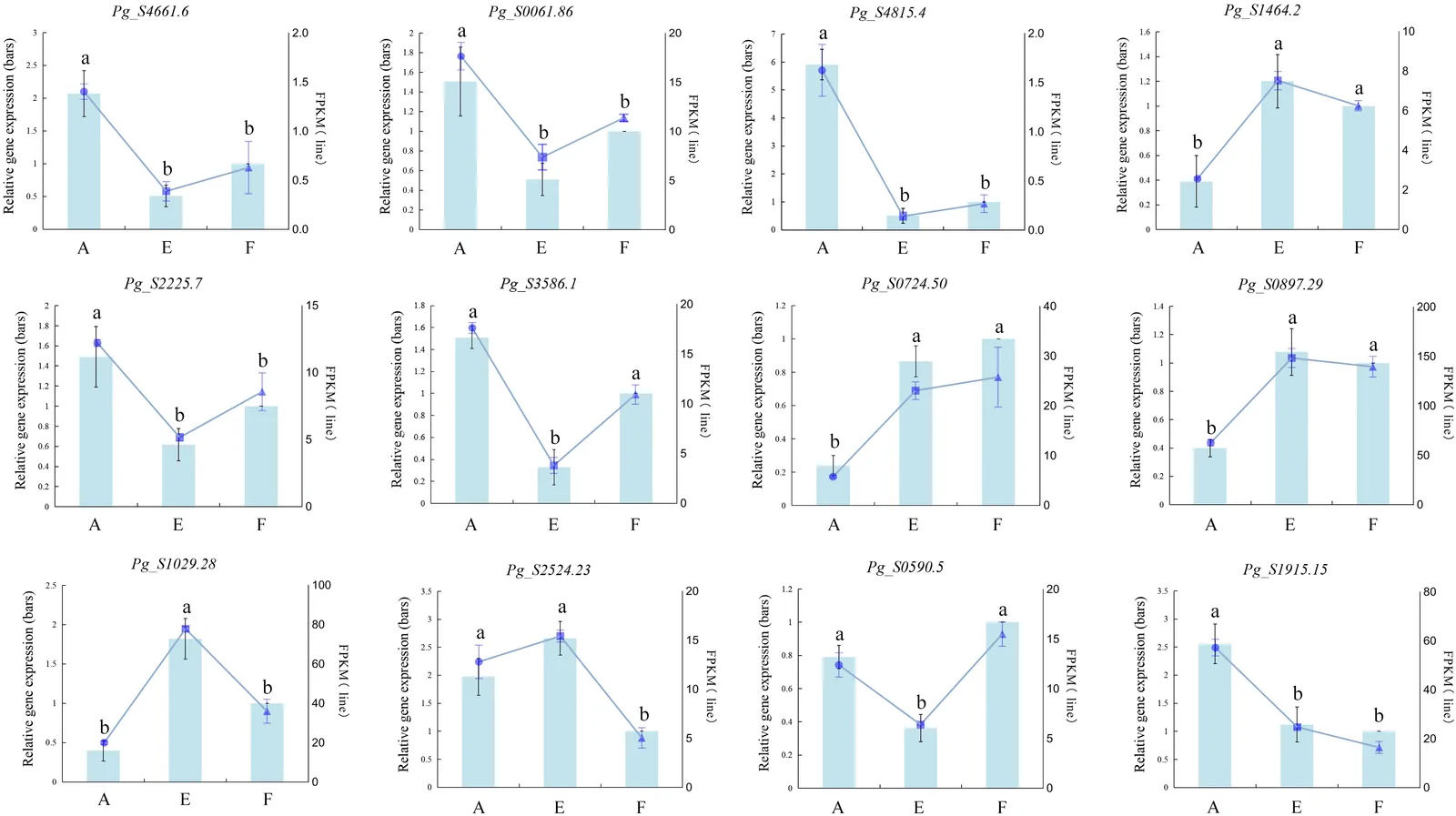
Results of the expression of 12 genes amplified by qRT-PCR. Significant differences among groups are denoted by distinct lowercase letters. A: sole nitrate; E: sole ammonium; F: nitrogen-free control.

## 4 Discussion

*Panax* ginseng is a traditional medicinal herb whose therapeutic value is primarily derived from ginsenosides, which are its core bioactive constituents. Although adventitious root culture has alleviated the pressure on wild ginseng resources to some extent, the efficiency of ginsenoside biosynthesis is a critical bottleneck that restricts industrial-scale production. In this study, the regulatory mechanisms by which nitrogen concentration and form affect ginsenoside accumulation were elucidated by combining nitrogen source manipulation with comparative transcriptomic analysis.

Nitrogen is an essential macronutrient that limits plant growth and development and directly influences the accumulation of secondary metabolites. The biomass and ginsenoside content of ginseng adventitious roots were lowest at total nitrogen of 5 mM, increased and peaked at 20 mM, with no further increase at nitrogen concentrations above 20 mM. There is therefore an optimal nitrogen concentration threshold for ginsenoside biosynthesis and accumulation, rather than a positive linear correlation with increasing nitrogen supply.

Based on the optimal total nitrogen of 20 mM, the NH_4_^+^:NO_3_^-^ ratio was adjusted. The nitrate-only treatment resulted in substantially higher adventitious root biomass and ginsenoside content than all other treatment groups, with the total ginsenoside content increasing by approximately 2.71-fold compared to the nitrogen-free control. With increasing ammonium concentration, both the biomass and ginsenoside content declined. This inhibitory effect of ammonium may be attributed to multiple physiological stresses induced by high NH_4_^+^ levels, including disruption of root cell membrane stability, disturbance of proton pump activity, and impairment of energy metabolism pathways (Zhu et al., 2021). These results are consistent with those of previous studies by Siwakumar et al (Sivakumar et al., 2005). and Geem et al. (2023), who reported that nitrate promoted ginseng growth and secondary metabolism, collectively indicating that nitrate and ammonium exerted distinctly different regulatory effects on cellular, tissue, and organ development, as well as on primary and secondary metabolism.

Using second-generation high-throughput sequencing,transcriptomic analysis of the ammonium-treated, nitrate-treated, and nitrogen-free control groups was conducted. GO functional annotation indicated that DEGs were enriched in metabolic regulation-related pathways under the biological process category, as well as in key enzyme classes, such as oxidoreductases, transferases, and hydrolases under the molecular function category. KEGG enrichment analysis indicated that the plant hormone signal transduction pathway was most enriched. These results suggest that different nitrogen sources may modulate phytohormone signal transduction, potentially triggering defense responses and promoting secondary metabolite accumulation as an adaptive response to nitrogen stress.

Ginsenoside biosynthesis involves multiple enzymatic steps, encompassing isoprenoid precursor supply, cyclization, and backbone modification (Jung et al., 2003; Liu et al., 2013; Seki et al., 2015; Song et al., 2019; Krishnamurthy et al., 2020; Hou et al., 2021). In the present study, nitrate treatment (A) upregulated HMGR in the MVA pathway and ispH in the MEP pathway, suggesting that nitrate may enhance precursor supply through both routes, whereas ammonium treatment (E) suppressed their expression. Downstream genes involved in precursor conversion and early cyclization—including GPS, FPS, SS, and SQE—as well as DDS and β-AS in the cyclization branch, all exhibited higher expression levels under nitrate treatment. Among these, DDS is the first committed enzyme in dammarane-type ginsenoside biosynthesis (Hou et al., 2021), and its expression changes may significantly affect total ginsenoside accumulation; this is supported by previous findings that DDS silencing led to an 84.5% reduction in ginsenoside content (Han et al., 2006; Lu et al., 2017). These transcriptional changes suggest that nitrate may activate the ginsenoside biosynthetic pathway at multiple levels. In addition to direct transcriptional regulation, nitrate assimilation may also enhance α-ketoglutarate production through carbon–nitrogen metabolic crosstalk, thereby increasing the carbon flux available for terpenoid precursor supply (Han et al., 2024). Collectively, these findings indicate that nitrate may promote ginsenoside accumulation by coordinately upregulating key enzyme genes and optimizing carbon–nitrogen metabolic flux, whereas ammonium exerts a broad suppressive effect on most key genes and metabolic processes within the pathway.

Transcriptome data indicated that, compared to ammonium-nitrogen, nitrate-nitrogen treatment upregulated NR and NiR gene expression and increased endogenous NO content. The NO scavenger, cPTIO eliminated the promotional effect of nitrate-nitrogen on biomass and ginsenoside accumulation, whereas exogenous SNP treatment restored this phenotype. These results suggest that nitrate-nitrogen assimilation promotes NO accumulation, and NO, as a key downstream signal, activates the transcription of structural genes involved in ginsenoside synthesis. Under ammonium-nitrogen conditions, the accumulation of NO was insufficient, which may partly explain the limited capacity of ammonium to promote saponin synthesis.

Cellular redox homeostasis constitutes another regulatory mechanism linking nitrogen form to secondary metabolism. Physiological and transcriptomic data revealed distinct antioxidant responses to nitrate and ammonium: SOD activity was higher under nitrate treatment, whereas ammonium supply induced higher CAT and POD activities and increased expression of their corresponding coding genes. Given that excess ammonium frequently triggers reactive oxygen species bursts and activates antioxidant defense systems, the altered redox state under ammonium may influence the activity of biosynthetic enzymes, while nitrate appears to maintain a relatively mild oxidative state conducive to continuous triterpenoid synthesis.

Phytohormones play central regulatory roles in plant growth, development, and environmental adaptation (Thilakarathne et al., 2025). Endogenous hormones are involved in plant growth and development, and mineral nutrient stress affects their synthesis and transport (Bo, 2022). Extensive crosstalk between phytohormones and nitrogen signaling has been well documented, and phytohormones have been reported to promote triterpenoid biosynthesis and accumulation by modulating key enzyme activities, regulating transcription factors, and facilitating synergistic interactions between different hormones (Cui et al., 2011; Lulu et al., 2015). In the present study, several key genes involved in hormone synthesis and signaling pathways, particularly those in the IAA and CTK pathways, exhibited substantial nitrogen form-dependent transcriptional responses, with most showing higher expression under nitrate treatment and lower expression under ammonium treatment.

CTK signaling exhibited differential responses to nitrogen form. Both endogenous CTK concentrations and transcript levels of the core signaling genes, HISTIDINE PHOSPHOTRANSFER PROTEIN (AHP) and TYPE-A RESPONSE REGULATOR (A-ARR), were higher under nitrate-only supply than under other treatments. Elevated CTK levels in nitrate-cultured adventitious roots may cooperate with IAA to establish a favorable hormonal balance, supporting continuous growth and secondary metabolism. Conversely, ammonium treatment reduced endogenous CTK content and restricted ginsenoside biosynthesis.

IAA plays important roles in cell elongation, differentiation, and tissue development (Zhao, 2018). Nitrogen deficiency induces the expression of IAA biosynthesis-related genes and accelerates IAA transport from shoots to roots (Bao et al., 2009). In the current study, endogenous IAA content was substantially higher under nitrate treatment than under ammonium or nitrogen-free conditions. Transcriptional analysis revealed that the Auxin Resistant 1 (AUX1)-encoding gene, *Pg-S0388.21*, was upregulated under nitrate but downregulated under ammonium. AUX1 is localized to the basal plasma membrane of root protophloem cells and facilitates polar IAA transport to pericycle cells, thereby promoting lateral root initiation (Swarup et al., 2001). Additionally, nine SMALL AUXIN UP-REGULATED RNA (SAUR)-encoding genes were upregulated following nitrate treatment. The SAUR gene family comprises IAA-responsive factors that promote cell expansion (Ren and Gray, 2015), and one DEG at the TRANSPORT INHIBITOR RESPONSE 1 (TIR1) node was also upregulated; TIR1 functions as an auxin receptor and plays a role in plant stress resistance (Tan et al., 2007).

Unlike the IAA and CTK pathways, GA pathway-related genes showed higher expression levels under ammonium and nitrogen-free treatments than under nitrate treatment. During GA signal transduction, one DEG associated with the GIBBERELLIN INSENSITIVE DWARF1 (GID1) node was identified. GID1 is a soluble nuclear receptor that perceives GA signals (Sun, 2008; Li et al., 2018).

Abscisic acid signaling plays an important role in root development and growth regulation. In the current study, most genes encoding PYRABACTIN RESISTANCE/PYR1-LIKE (PYR/PYL) —the ABA receptors—exhibited higher expression under ammonium supply and nitrogen-free conditions than under nitrate supply, indicating that ammonium stress and nitrogen limitation activate ABA-mediated stress responses, whereas nitrate-grown adventitious roots maintain relatively low ABA signaling activity. Ondzighi-Assoume et al. (2016) demonstrated that nitrate regulates root growth by modulating ABA accumulation at the root tip, as well as ethylene and brassinosteroid signaling. Nitrate availability also affects ethylene synthesis and transport (Tian et al., 2009), and brassinosteroid signaling is involved in root growth regulation under low-nitrogen stress in *Arabidopsis* (Jia et al., 2019).

In summary, nitrate activates IAA and CTK signaling pathways, whereas GA pathway-related genes show higher expression under ammonium and nitrogen-free conditions, together forming a nitrogen-responsive hormonal regulatory network associated with ginsenoside accumulation. The hormonal mechanisms by which nitrogen regulates plant secondary metabolism were supported by qRT-PCR validation. Although NO was validated as a key signal, the molecular cascade connecting NO to ginsenoside biosynthesis genes remains uncharacterized. Future investigations of NO-regulated transcription factors and their crosstalk with hormone and redox pathways will further elucidate the nitrogen signaling network that controls secondary metabolism in *P. ginseng.*

## 5 Conclusion

In this study, the effects of nitrogen forms and ammonium nitrate ratio on the accumulation of ginsenosides in ginseng roots were systematically investigated, and the optimal total nitrogen concentration was 20 mM. Compared with ammonium nitrogen treatment, nitrate nitrogen treatment obtained better biomass and ginsenoside accumulation. Transcriptome data showed that nitrate up-regulated a series of core genes involved in ginsenoside biosynthesis, remodeled plant hormone homeostasis and antioxidant defense, and triggered endogenous NO production. Further experiments confirmed that NO acts as an important signal to transmit nitrate signals, regulating root growth and triterpene biosynthesis. This work reveals the molecular mechanism of nitrogen form-dependent ginsenoside formation and provides guidance for the optimization of nitrogen strategy in ginseng adventitious root culture.

## Data Availability

The datasets presented in this study can be found in online repositories. The names of the repository/repositories and accession number(s) can be found in the article/**Supplementary Material**.
