# Supplementary material for: Nitrogen form regulates ginsenoside biosynthesis in Panax ginseng adventitious roots: comparative transcriptomics of ammonium- and nitrate-mediated pathways
Source: Front Plant Sci. 2026 Sep 28;17:1948127. doi: 10.3389/fpls.2026.1948127 (PMC13655007; doi:10.3389/fpls.2026.1948127)
Supplement: Supplementary file 1 [file DataSheet1.doc]

Figure S1：Differentially expressed genes GO annotation classification statistics. Note: A: sole nitrate; E: sole ammonium; F: nitrogen-free control;

Figure S2: PCA of transcriptome profiles of *Panax ginseng* adventitious roots. Note: A: sole nitrate; E: sole ammonium; F: nitrogen-free control;
